# Serotonergic SLC10A4 regulates extracellular serotonin levels and antidepressant responsiveness

**DOI:** 10.1038/s41380-026-03697-y

**Published:** 2026-06-30

**Authors:** Fabio V. Caixeta, Stina Lundberg, Akilandeswari Balasubramanian, Charlotte van Gelder, Ella Mercer, Guofeng Lou, Jörgen Jonsson, Åsa Konradsson-Geuken, Klas Kullander

**Affiliations:** 1 Department of Immunology, Genetics & Pathology, Uppsala University, Uppsala, Sweden; 2 Indian Institute of Science Education and Research, Pune, Pune, 411008 India; 3 Section of Neuropharmacology and Addiction Research, Department of Pharmaceutical Biosciences, Uppsala University, Uppsala, Sweden; 4 Present Address: Department of Physiological Sciences, University of Brasília, Brasília, Brazil; 5 Present Address: Department of Medical Cell Biology, Uppsala University, Uppsala, Sweden; 6 Present Address: Biohub, Redwood City, CA 94063 USA; 7 Present Address: Center for Molecular Medicine & Oncode Institute, University Medical Center Utrecht, Utrecht University, 3584 CG Utrecht, the Netherlands; 8 Present Address: The Tuberous Sclerosis Association, Edinburgh, UK; 9 Present Address: Fosun Pharma Europe, Frankfurt, Germany; 10 Present Address: Functional Pharmacology and Neuroscience, Department of Surgical Sciences, Uppsala University, Uppsala, Sweden

**Keywords:** Neuroscience, Molecular biology

## Abstract

Solute carrier family member 10A4 (SLC10A4) is an orphan transporter selectively expressed in aminergic cells, where it localizes to synaptic vesicles and has been implicated in aminergic neurotransmission. Although constitutive knockout studies have suggested a role for SLC10A4 in monoamine and acetylcholine homeostasis, interpretation has been limited by developmental compensation and lack of cell-type specificity. Here, we employed a tamoxifen-inducible CreERT2–lox system to selectively delete *Slc10a4* in serotonergic neurons of young adult mice (8-10 weeks), enabling assessment of its function in mature serotonergic circuits. Adult serotonergic deletion of SLC10A4 resulted in a marked reduction of baseline extracellular serotonin levels in the medial prefrontal cortex, as measured by in vivo microdialysis. Notably, the ability of the selective serotonin reuptake inhibitor fluoxetine to elevate extracellular serotonin was strongly attenuated. These neurochemical deficits were accompanied by increased anxiety-like behavior across multiple behavioral paradigms and a blunted antidepressant-like response to fluoxetine in the forced swim test, while locomotor activity and motor coordination remained intact. These findings show that SLC10A4 is required for normal extracellular serotonin availability and antidepressant responsiveness in the adult brain. Together, our results identify SLC10A4 as a novel critical regulator of serotonergic neurotransmission and suggest that presynaptic monoamine handling represents an important and previously underappreciated determinant of affective behavior and treatment efficacy.

## Introduction

The solute carrier (SLC) superfamily comprises 76 gene families encompassing more than 450 functional protein-coding genes, making it one of the largest transporter superfamilies in humans [1]. Although SLC transporters are essential for cellular homeostasis [2] and are implicated in more than 80 monogenic diseases, the SLC family remains among the least studied groups of human genes [3]. Notably, nearly 25% of SLCs are still classified as orphans, proteins with unknown or incompletely characterized functions, highlighting their considerable potential as therapeutic targets [1].

The SLC10 family, initially thought to function primarily in bile acid transport, is now recognized to mediate a broad range of substrates, including steroid hormones, drugs, and other molecules [4, 5]. To date, only three of the seven members of this family (Slc10a1, Slc10a2 and Slc10a6) have been deorphanized, and recent evidence indicates that hitherto orphan members are involved in diverse cellular processes, including apoptosis [6], calcium-mediated glycosylation in the Golgi apparatus [7], and mast cell degranulation [8].

SLC10A4 is an orphan transporter [9] that, in the central nervous system, co-localizes with synaptic vesicle proteins, including the vesicular acetylcholine transporter (VAChT/*Slc18a3*) and vesicular monoamine transporter 2 (VMAT2/*Slc18a2*), leading to its suggested designation as vesicular aminergic-associated transporter, or VAAT [10]. It is expressed in the human brain in a pattern similar to that observed in rodents, and reduced SLC10A4 expression in the trans-entorhinal cortex correlates with the severity of Alzheimer’s disease–related pathology [11]. In rodents, SLC10A4 deficiency produces mild impairments in spatial memory and cognitive flexibility [12], altered sensory gating [13], and heightened sensitivity to both the cholinergic chemoconvulsant pilocarpine [14] and the dopaminergic psychostimulant amphetamine [10]. Moreover, synaptic vesicles isolated from *Slc10a4* knockout (KO) mice exhibit reduced monoamine content and impaired dopamine uptake [10], suggesting a regulatory role for SLC10A4 in aminergic neurotransmitter homeostasis. These findings led to the hypothesis that SLC10A4 contributes to vesicle sorting and filling of monoamine transmitters [10, 15]. However, no study has yet examined the function of SLC10A4 within specific aminergic nuclei, nor has any addressed the confounding influence of developmental compensation in constitutive knockout models.

The serotonin system is a long-standing target for the treatment of mood disorders [16, 17]. Genetic alterations in serotonergic signaling have been linked to major depressive disorder [18, 19], anxiety [20, 21], increased stress susceptibility, irritable bowel syndrome [19], obsessive-compulsive disorder [22], and suicide [23].

Here, we selectively deleted *Slc10a4* in serotonergic cells of adult mice to determine the role of SLC10A4 in serotonergic neurotransmission and behavior. To avoid developmental compensation observed in constitutive knockout models, we used a tamoxifen-inducible CreERT2-lox system to restrict gene deletion spatiotemporally to serotonergic neurons in adulthood. Based on previous findings showing that loss of SLC10A4 reduces monoamine levels [10], we hypothesized that serotonergic-specific deletion of *Slc10a4* would lead to increased anxiety- and depression-related behaviors.

## Materials and methods

### Transgenic animals

All experimental animals were bred, housed, and tested at the animal care facility of Uppsala University under ethical permits C144/12, C135/14, and C43/16, following approval by the Uppsala Animal Ethical Committee. Mice were weaned and genotyped at 4 weeks of age and housed in groups of 2–5 littermates, with at least one experimental and one control animal per cage, under a 12-h light/dark cycle (lights on at 07:00). Littermates were allocated to experimental groups based on genotype after weaning. No formal randomization was used. Behavioral testing order was standardized across cohorts. Food and water were provided *ad libitum*.

**Fig. 1:**
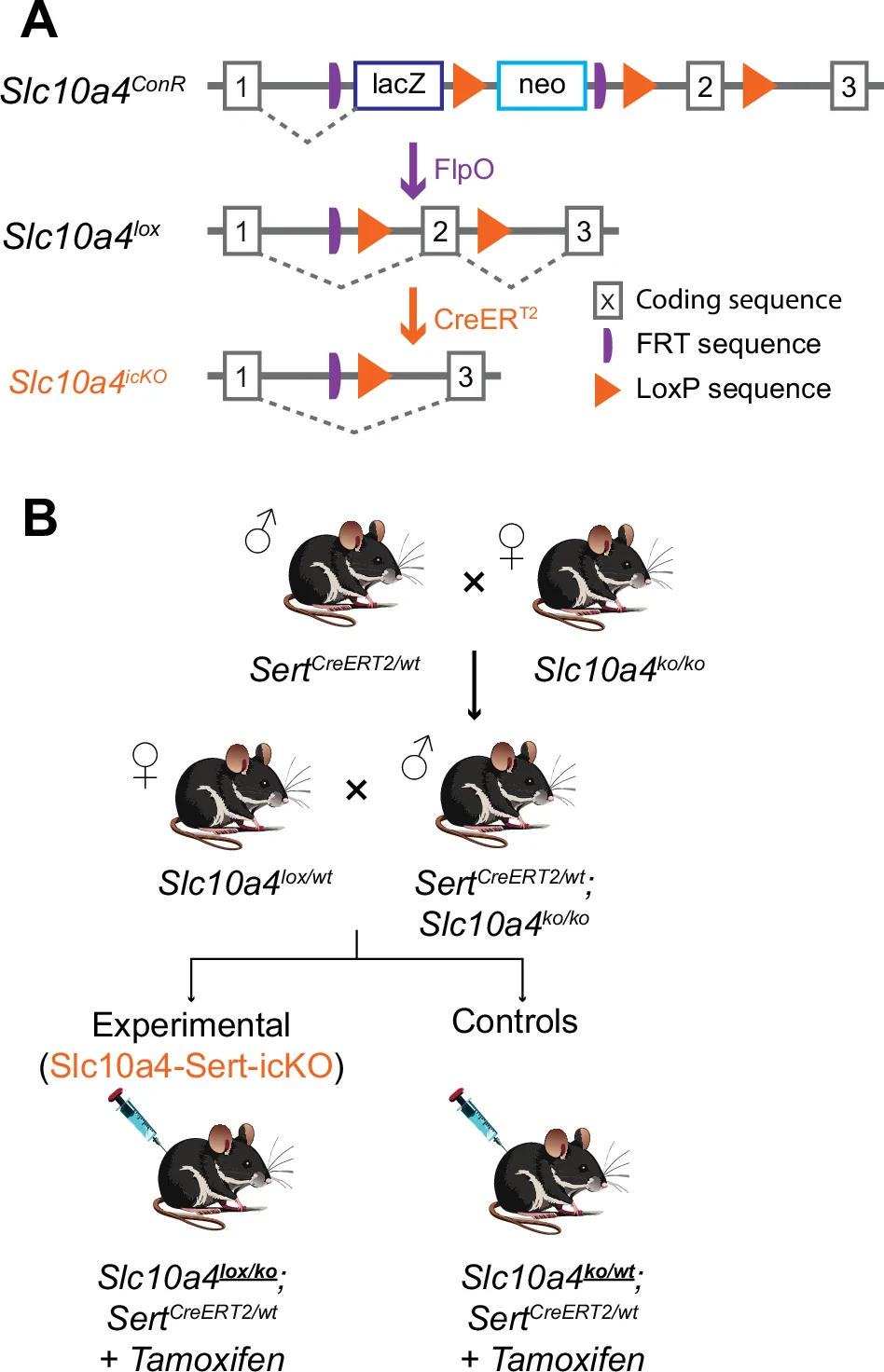
Generation of the inducible, serotonergic-specific *Slc10a4* knockout mouse line Slc10a4-Sert-icKO. **A** Schematic illustration of the genetic strategy used to convert the conditional-ready *Slc10a4* allele (*Slc10a4*^*ConR*^) into a Cre-dependent knockout allele (*Slc10a4*^*icKO*^) via FLPo- and CreER^T2^-mediated recombination (modified from Skarnes et al., 2011). **B** Breeding strategy used to generate experimental and control mice.

To generate the inducible and conditional knockout line (Fig. 1A), a loxP-flanked *Slc10a4* allele (*Slc10a4*^*lox*^) was produced by crossing mice carrying the conditional-ready *Slc10a4*^*tm1a(KOMP)Wtsi*^ (*Slc10a4*^ConR^) allele from the Knockout Mouse Project (KOMP, Davis, CA, USA) with the FLPo deleter line B6.129S4-*Gt(ROSA)-26Sor*^tm2(FLP*)Sor/J^ (Jackson Lab, Bar Harbor, ME, USA). The resulting mice were subsequently backcrossed to C57BL/6 J mice (Jackson Laboratory) to remove the FLPo allele and minimize unwanted genetic variability.

For time-controlled deletion restricted to serotonergic neurons, we used the inducible Cre line B6.FVB(Cg)-Tg(Slc6a4-Cre/ERT2)-EZ13Gsat/Mmucd (RRID: MMRRC_032109-UCD; *Sert*^*CreERT2/wt*^) obtained from the Mutant Mouse Resource & Research Centers (MMRRC, Davis, CA, USA). In this line, the CreERT2 recombinase is expressed under the control of the serotonin transporter (*Slc6a4*, or *Sert*) promoter, and is activated by tamoxifen with minimal baseline recombination [24]. In addition, *Slc10a4* null mice (*Slc10a4*^*ko/ko*^) generated by the Texas A&M Institute for Genomic Medicine (TIGM, College Station, TX, USA) as previously described [10] and the reporter line tdTomato (*Gt(ROSA)26Sor*^tm14(CAG-tdTomato)Hze^; or simply *tdTomato*^*tg*^; Allen Brain Institute, Seattle, WA, USA) were used where indicated. All mouse lines were maintained on a C57BL/6 background, either by the supplier or by backcrossing for at least 10 generations in our facility.

Genotyping was performed by PCR on ear biopsy DNA using primers provided by the suppliers or designed with Primer3Plus [25]. PCR reactions were carried out using KAPA Taq (KAPA Biosystems, Cape Town, South Africa) or Maxima Hot Start Taq DNA Polymerase (Thermo Fisher Scientific, Vilnius, Lithuania).

### Tamoxifen treatment

To induce CreERT2 activity in serotonergic neurons, mice received tamoxifen (Sigma-Aldrich, Wuxi, China) at a dose of 2 mg per day, dissolved in 0.1 mL of a 90% sunflower oil / 10% ethanol solution, administered via intraperitoneal (i.p.) injections once daily for three consecutive days (total dose 6 mg per animal) at 8–10 weeks of age (young adulthood). Mice used for immunostaining and in situ hybridization were euthanized 1 week after tamoxifen administration, corresponding to approximately 9–11 weeks of age, whereas behavior experiments were performed 3 weeks after tamoxifen treatment.

### Tissue preparation for histology

Adult mice were deeply anesthetized and perfused transcardially with phosphate-buffered saline (PBS; Sigma-Aldrich, Laramie, WY, USA), followed by 4% formaldehyde in PBS. Brains and spinal cords were dissected and post-fixed overnight at 4 °C in 4% formaldehyde.

Spinal cords were subsequently washed in PBS and cryoprotected in PBS containing 30% sucrose for 24 h at 4 °C, embedded in Neg-50™ Frozen Section Medium (Thermo Fisher Scientific, Kalamazoo, MI, USA), rapidly frozen on dry ice, and stored at −80 °C until cryosectioning. Cryosections (16 µm) were collected using a cryostat (Cryocut 1800, Leica) onto Superfrost Plus slides (Thermo Fisher Scientific) and stored at -20 °C.

Brain tissue, after post-fixation, was embedded in 4% agarose and sectioned immediately at 60 µm using a vibratome (VT1200, Leica).

### In situ hybridization

For RNA in situ hybridization, we used a digoxigenin (DIG)-labeled antisense riboprobe targeting nucleotides 1375–2085 of the *Slc10a4* mRNA (NCBI accession number NM_173403.2), corresponding to a ~ 710 bp region within the coding sequence. A plasmid containing *Slc10a4* cDNA was linearized with HincII (Promega, Madison, WI, USA), and complete digestion was confirmed by agarose gel electrophoresis.

DIG-labeled antisense riboprobes (Roche, Mannheim, Germany) were synthesized by in vitro transcription using 1.5 µg of linearized template and the T3 Polymerase. The resulting probes were diluted in nuclease-free water, aliquoted (20 µL), stored at −20 °C, and their integrity verified on a 2% agarose gel.

On day 1, cryosections were washed in PBS, fixed in 4% formaldehyde for 10 min, and treated with Proteinase K (6 µg/mL) for 8 min, followed by postfixation and acetylation. Sections were then pre-incubated in hybridization buffer (50% formamide, 5× Denhardt’s solution, 50 µg/mL salmon sperm DNA, and 50 µg/mL yeast tRNA) for 2 h at room temperature. The DIG-labeled antisense riboprobe was denatured and applied to sections at a final concentration of 300 ng/mL in hybridization buffer (150 µL per section), coverslipped, and incubated for 16–20 h at 55 °C.

On day 2, slides were washed in 5× SSC for 5 min, followed by stringency washes in 0.2× SSC at 55 °C for 1 h and an additional wash in 0.2× SSC for 30 min at room temperature. For probe detection, sections were blocked for 2 h in blocking solution (5% goat serum, 0.3% Triton X-100 in TBS) and incubated overnight at 4 °C with anti-DIG alkaline phosphatase–conjugated Fab fragments (1:1000; Roche) diluted in blocking solution.

On day 3, sections were washed three times for 15 min in TBST (0.3% Triton X-100 in TBS) and once for 15 min in TBST containing 2 mM levamisole. Sections were equilibrated in NTMT buffer for 10 min before color development with BM Purple (Roche) at 37 °C. The reaction was stopped after 4 h by extensive washing in PBS. Slides were mounted with Mowiol® mounting medium, and images were acquired using a confocal microscope (Zeiss LSM 510 META).

### Immunofluorescence staining

Vibratome-sectioned brain slices were washed in PBS and pre-blocked for 90 min in blocking solution (2% bovine serum albumin, 6% goat serum, and 0.3% Triton X-100 in PBS), followed by overnight incubation at 4 °C with primary antibodies diluted in blocking solution. Primary antibodies against SLC10A4 (Sigma, HP028835), VAChT (Millipore, AB1588), NeuN (Millipore, MAB3777), and VMAT2 (Santa Cruz, sc-390285) were used.

After primary antibody incubation, sections were washed three times for 30 min in blocking solution and incubated with Alexa Fluor–conjugated secondary antibodies (488, 594, or 647; 1:1000) and DAPI (1:1500), diluted in blocking solution, for 2 h at room temperature or overnight at 4 °C. Sections were then washed twice for 30 min in blocking solution, three times for 15 min in PBST (0.3% Triton X-100 in PBS), and twice for 10 min in PBS. Slices were mounted with Mowiol® 4-88 (Sigma-Aldrich), and images were acquired using either a confocal microscope or a fluorescence microscope (Olympus BX61WI).

All images were adjusted for brightness and contrast using GIMP. All datasets used for genotype comparisons were acquired under identical imaging settings. For manual cell counting, 60-µm-thick brain sections containing the raphe nuclei were analyzed within a 100 × 500 µm region of interest spanning the midline of the dorsal raphe nucleus.

### In vivo microdialysis

In vivo microdialysis was used to measure extracellular serotonin levels in the medial prefrontal cortex under baseline conditions and following fluoxetine administration. Adult mice (>8 weeks, 25–32 g) were anesthetized with isoflurane and mounted in a stereotaxic frame. A CMA-7 guide cannula (CMA Microdialysis AB, Sweden) was implanted in the medial prefrontal cortex (mPFC; AP + 1.94 mm, ML − 0.37 mm, DV − 2.25 mm) and secured with dental cement (Tetric EvoFlow, Ivoclar Vivadent, Switzerland).

Seven days later, animals were anesthetized with isoflurane and a CMA-7 probe was inserted and perfused with physiological solution (in mM: 148.0 NaCl, 4.0 KCl, 0.8 MgCl2, 1.4 CaCl2, 1.2 Na2HPO4, 0.3 NaH2PO4, pH 7.2) at 1.0 µL/min with a CMA-100 infusion pump (CMA Microdialysis AB, Sweden). Samples of the extracellular fluid were manually collected every 20 min and diluted (2:1) with phosphate buffer (0.1 M with pH, 3.0 plus 0.1 mM EDTA-2Na) and kept on dry-ice until the end of the experiment. After a 120-min baseline, mice received fluoxetine (20 mg/kg, i.p.), and sampling continued for 180 min.

After the experiment, each mouse was euthanized and the brain was dissected and post-fixed in 4% PFA solution for 24 h at 4 °C. 50 µm thick sections of the brain were prepared using a vibratome (VT1000S, Leica, Germany) and verified for probe placement using the Mouse Brain Stereotaxic Atlas [26].

Dialysate samples were analyzed by using high performance liquid chromatography with electrochemical detection (HPLC-EC System, ESA, USA). The mobile phase consisted of 75 mmol/L NaH2PO4, 1.4 mmol/L sodium octyl sulphate and 10µmol/L EDTA in deionized water containing 7% acetonitrile brought to pH 3.1 with phosphoric acid. Serotonin levels in dialysate samples were calculated (Clarity™ Software, DataApex Ltd, Prague, Czech Republic) by measurement of peak heights relative to known amounts in external standard serotonin (2 ng/mL, Sigma, USA).

### Behavioral tests

Behavioral experiments were conducted in male mice 3 weeks after tamoxifen administration. All behavioral tests were performed by a single experimenter, while tamoxifen and fluoxetine injections were administered by a second experimenter. Both experimenters were blinded to genotype during testing and data analysis. Animals were acclimated to the testing room for at least 1 h prior to testing. All tests were performed in the afternoon unless otherwise stated.

Behavioral experiments were conducted exclusively in male mice to minimize variability associated with estrous cycle–dependent hormonal fluctuations. While this approach reduces within-group variability, it limits the generalizability of the findings across sexes. Animals were derived from 12 independent litters and grouped into five cohorts at weaning. Variation in sample size across tests reflects that not all cohorts completed the full behavioral battery.

The behavioral battery was performed in the following order, with intervals of at least 3 days between tests to minimize potential carryover effects: elevated plus maze (EPM), open field (OF), novelty-suppressed feeding (NSF), forced swim test (FST), marble burying test (MBT), beam walk and challenging beam walk (BW and CBW).

The elevated plus maze consisted of two open arms (35 × 5.5 cm) with a 2-mm ledge, two closed arms (35 × 5.5 × 15 cm), and a central platform (5.5 × 5.5 cm). Illumination in the center was 100 ± 2 lux. Mice were placed in the center and allowed to explore for 5 min. Locomotor activity was recorded using EthoVision XT (Noldus, Netherlands). Primary outcome measures included time spent and speed of movement in the open arms of the apparatus and head-dipping behavior.

For novelty-suppressed feeding, mice were food-deprived for 12 h during the dark phase. A food pellet was placed on a platform in the center of a 30-cm circular arena. Mice were given up to 10 min to begin feeding. Latency to the first bite was recorded, with a maximum score of 600 s. Latency to enter the platform, contact the food pellet and to feed were used as anxiety-related outcome measures.

For the marble-burying test, mice were placed in cages (30 × 45 cm) filled with 5 cm of compacted bedding, with 10 marbles arranged in two rows. Animals were allowed to explore for 20 min (100 ± 2lux). A marble was considered buried if ≥ two-thirds of its surface was covered. The number of marbles buried was used as the primary outcome measure.

For the forced swim test, mice were placed in a transparent cylinder (30 cm diameter, 20 cm water depth, 25 ± 1 °C). On day 1, mice were recorded for 10 min. On day 2, mice received fluoxetine (20 mg/kg, i.p.) 30 min prior to testing and were recorded for 6 min, with the final 4 min scored. After testing, mice were warmed and dried. Fluoxetine was dissolved in 10% DMSO and 90% saline. Immobility duration during the final 4 min of the test was used as the primary measure of behavioral despair.

For the open-field test, mice were placed in the center of a 50 × 50 × 50 cm transparent arena (250 ± 5lux) for 20 min. The center zone was defined as the central 25 × 25 cm area. Locomotion was recorded using EthoVision XT. Primary outcome measures included total distance traveled, average speed and distance traveled in the center zone.

The beam-walk task consisted of crossing a 1-m beam positioned 35 cm above the floor to reach the home cage. The beam had five progressively narrower segments (60, 48, 36, 24, and 12 mm). Hind-limb slips were counted. Mice were trained for two days prior to testing. The challenging beam walk was identical but included a wire mesh overlay. Both were performed on the same day. The primary outcome measure was the number of foot slips.

### Statistical analysis

Statistical analyses were performed using Statistica 14.2 (TIBCO Software Inc.). Sample sizes were determined based on prior studies using comparable experimental paradigms and expected effect sizes; no formal a priori power calculation was performed. Animals were included based on successful genotyping and completion of the relevant experimental protocol. For microdialysis, only animals with verified probe placement in the mPFC were included. No additional exclusion criteria were applied. Normality was examined using Shapiro-Wilk’s W test, which guided the selection of subsequent statistical tests.

Immunofluorescence quantification, behavioral data, and normalized microdialysis baseline values were largely non-normally distributed and thus analyzed using nonparametric statistics. Single-point measures were analyzed with the Mann-Whitney U-test (MW, for two groups) or the Kruskal-Wallis ANOVA by ranks (KW), with MW post hoc tests when appropriate (for more than two groups).

For within-group paired comparisons involving two repeated time points, Wilcoxon matched-pairs signed-rank tests were used. The microdialysis time-course data was normally distributed and analyzed with a repeated measures ANOVA with post hoc Tukey HSD test when appropriate. Forced swim test data were analyzed using a two-way repeated measures ANOVA with genotype as a between-subject factor and treatment (baseline vs fluoxetine) as a within-subject factor. Categorical data was analyzed with Maximum-likelihood Chi-square tests.

## Results

### Inducible serotonergic deletion of *Slc10a4* in adult mice

To investigate the contribution of SLC10A4 to serotonergic function while minimizing compensatory effects during development, we designed a genetic strategy enabling spatially and temporally controlled deletion of *Slc10a4*. Experimental mice were generated by combining a conditional *Slc10a4* allele with a tamoxifen-inducible serotonergic Cre driver, yielding *Slc10a4*^*lox/ko*^*; Sert*^*CreERT2/wt*^ animals (Fig. 1A, B), hereafter referred to as *Slc10a4*-Sert-icKO mice.

The *Slc10a4*^*lox/ko*^ genotype was chosen instead of *Slc10a4*^*lox/lox*^ to ensure complete gene deletion following recombination, as heterozygous deletion of *Slc10a4* does not produce detectable phenotypes [10]. Control mice carried the genotype *Slc10a4*^*ko/wt*^*; Sert*^*CreERT2/wt*^ and differed from experimental animals only by the absence of the conditional *Slc10a4*^*lox*^ allele. Consequently, tamoxifen treatment selectively abolished *Slc10a4* expression in serotonergic neurons of experimental but not control mice.

The conditional *Slc10a4* allele was generated using the “conditional-ready” gene targeting strategy [27]. Because SLC10A4 is robustly expressed in cholinergic motor neurons of the spinal cord, where it co-localizes with VAChT, this region provided a reliable system to validate the functionality of the genetic constructs, including the conditional-ready (‘ConR’) *Slc10a4* allele, in which a gene-trap cassette disrupts endogenous *Slc10a4* expression (Fig. 1A).

**Fig. 2:**
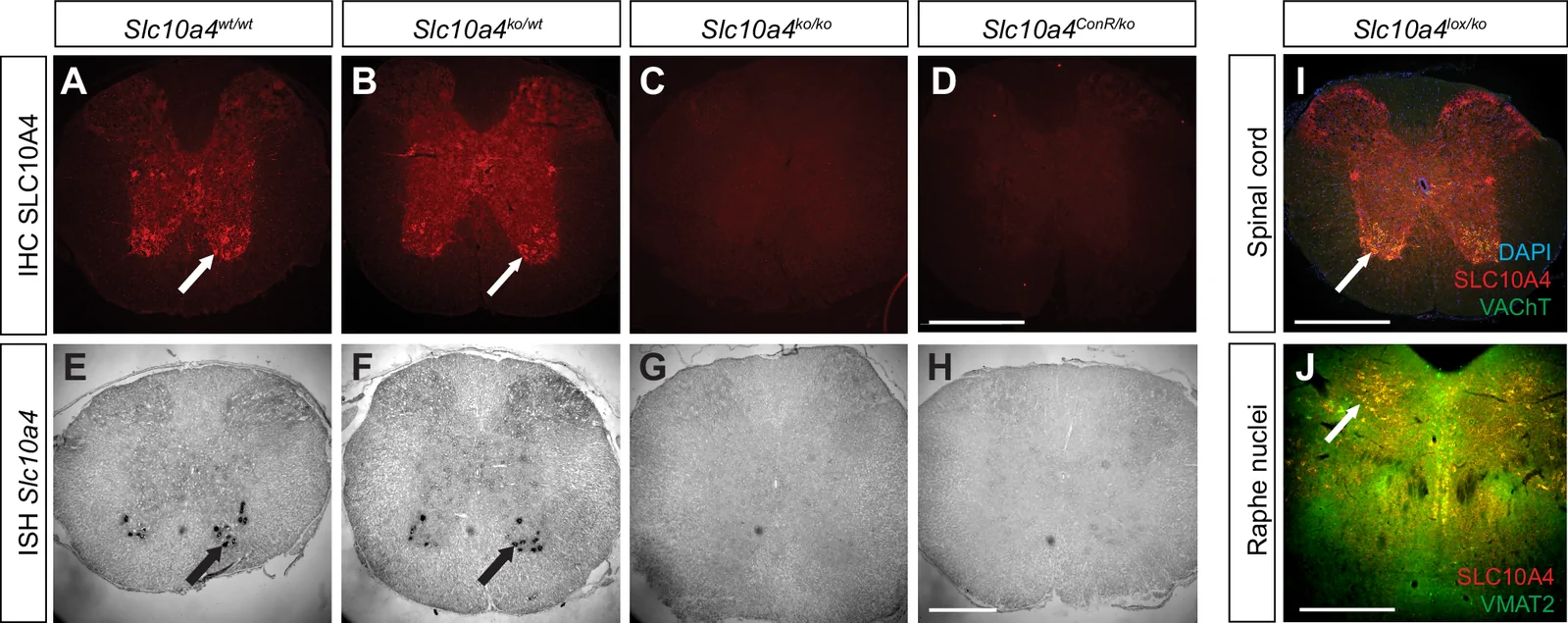
Validation of the conditional *Slc10a4* knockout strategy. **A**–**D** Immunohistochemical (IHC) detection of SLC10A4 protein in coronal spinal cord sections from mice of different *Slc10a4* genotypes (above each panel), showing expression in ventral motor neurons (arrows). **E**–**H** In situ hybridization (ISH) detecting *Slc10a4* mRNA in corresponding spinal cord sections, confirming genotype-dependent loss of transcript in ventral motor neurons (arrows). **I**, **J** Immunofluorescence demonstrating preserved SLC10A4 expression from the conditional *Slc10a4*^*lox*^ allele in cholinergic neurons of the spinal cord (**I**; co-labeled with VAChT) and in serotonergic neurons of the dorsal raphe nucleus (**J**; co-labeled with VMAT2). Scale bars: 600 µm (**A**–**D**, **I**, **J**) and 400 µm (**E**–**H**).

Immunofluorescence analysis of spinal cord sections revealed strong SLC10A4 expression in ventral motor neurons of mice carrying at least one wild-type allele (*Slc10a4*^*wt/wt*^ and *Slc10a4*^*ko/wt*^; Fig. 2A, B). In contrast, SLC10A4 immunoreactivity was completely absent in both *Slc10a4*^*ko/ko*^ and *Slc10a4*^*ConR/ko*^ animals (Fig. 2C, D). RNA in situ hybridization confirmed the absence of *Slc10a4* transcripts in these genotypes (Fig. 2E–H), demonstrating that the *Slc10a4*^*ConR*^ allele functions as a null allele equivalent to the constitutive knockout. To generate the final conditional allele, *Slc10a4*^*ConR*^ mice were crossed with FLPo deleter mice, resulting in excision of the gene-trap cassette and production of the *Slc10a4*^*lox*^ allele (Fig. 1A). FLPo is ubiquitously expressed from early embryonic stages [28] for recombination in all tissues, including the germ line.

Importantly, immunofluorescence analysis confirmed that SLC10A4 expression was preserved in *Slc10a4*^*lox*^ mice. SLC10A4 co-localized with VAChT in spinal motor neurons (Fig. 2I) and with VMAT2 in serotonergic neurons of the dorsal raphe nucleus (Fig. 2J), consistent with the endogenous aminergic expression pattern of SLC10A4 [10].

**Fig. 3:**
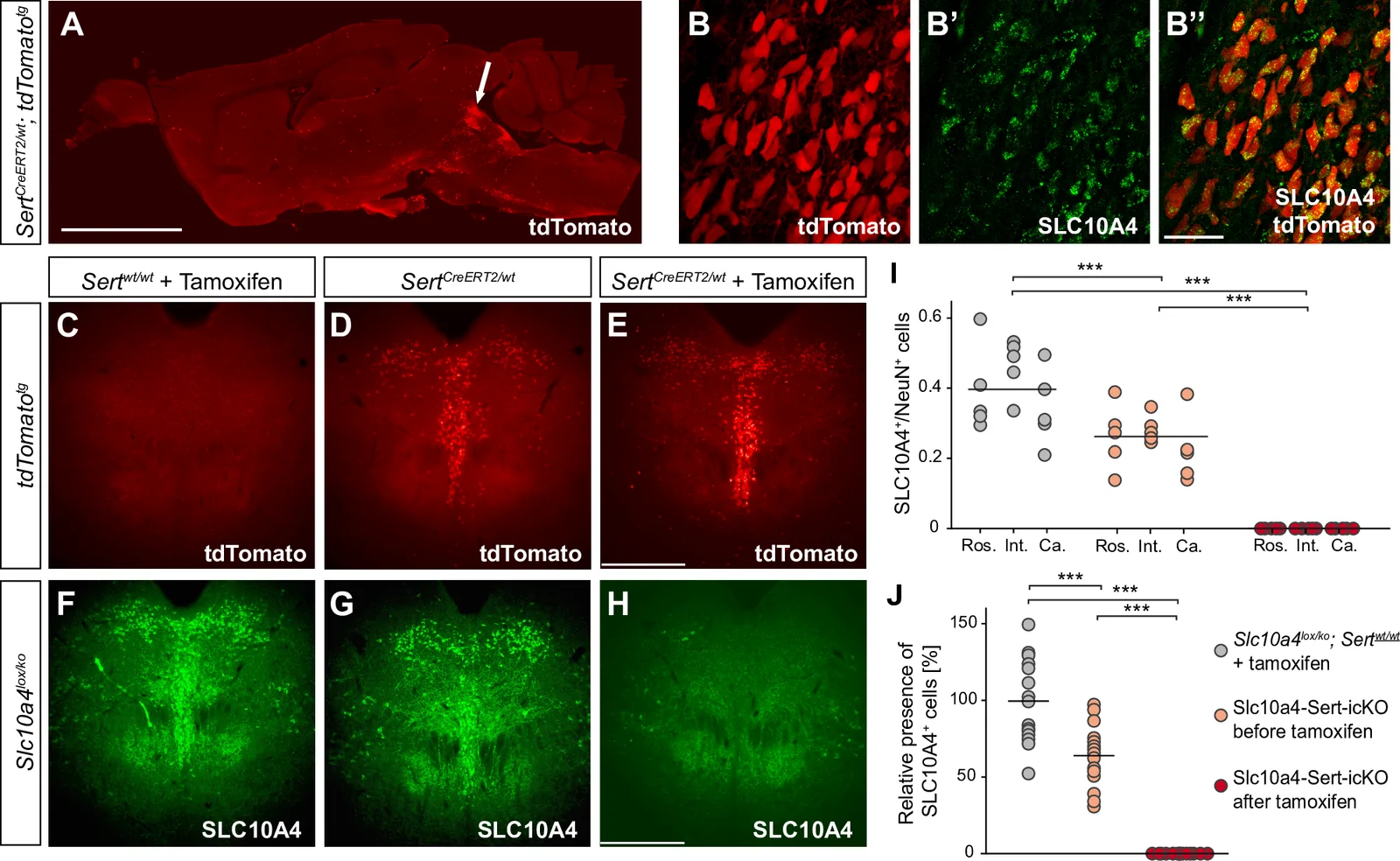
Conditional deletion of *Slc10a4* in serotonergic neurons of the dorsal raphe nucleus of Slc10a4-Sert-icKO mice. **A** Sagittal brain section from a *Sert*^*CreERT2/wt*^; *tdTomato*^*tg*^ mouse showing *tdTomato* expression in the rostral cluster of raphe nuclei (arrow). **B** Immunofluorescence illustrating vesicular *Slc10a4* expression in tdTomato-positive serotonergic neurons of the dorsal raphe; single-channel images for tdTomato (**B**), SLC10A4 (**B**’) and the merged image (**B**”) are shown. **C**–**E** tdTomato fluorescence in the dorsal raphe nuclei of *Slc10a4*^*lox/ko*^ mice lacking *CreERT2* (**C**), expressing *CreERT2* without tamoxifen treatment, also known as ‘leaky-Cre expression’ (**D**), or expressing *CreERT2* following tamoxifen administration (**E**). **F**–**H** Corresponding SLC10A4 immunostaining in the same genotypes and conditions as in panels **C**–**E**, demonstrating efficient loss of *Slc10a4* expression following tamoxifen-induced recombination. **I**−**J** Quantification analysis of either the ratio of SLC10A4^+^/NeuN^+^ cells (**I**), or relative presence of SLC10A4 positive cells (**J**), confirms that SLC10A4 expression was only fully abolished after tamoxifen treatment. Rostral (Ros.), intermediate (Int.) and caudal (Ca.) subregions of the dorsal raphe nucleus are shown for Cre-negative control mice (gray) and in Slc10a4-Sert-icKO mice before (orange) or after (red) tamoxifen treatment (individual values with median; ***p < 0.001; Kruskal-Wallis ANOVA by ranks, with Mann-Whitney U post hoc tests). The phenotype of the Slc10a4-Sert-icKO mouse is represented in panel **H**, and quantitatively in the rightmost columns in panels **I** and **J**. Scale bars: 1 mm (**A**), 30 µm (**B**), and 600 µm (**C**–**H**).

To achieve inducible deletion of *Slc10a4* specifically in serotonergic neurons, we used the Sert^CreERT2^ driver line, in which CreERT2 expression is controlled by the serotonin transporter promoter. To characterize recombination specificity and efficiency, SertCre^ERT2^ mice were crossed with tdTomato reporter animals. Following tamoxifen administration, robust tdTomato expression was observed in major serotonergic nuclei, particularly within the dorsal and median raphe (Fig. 3A), and tdTomato-positive cells co-localized with SLC10A4 immunoreactivity (Fig. 3B). These results confirm that *Sert*^*CreERT2*^ effectively targets serotonergic neurons.

Low levels of tdTomato fluorescence were also detectable in the raphe nuclei prior to tamoxifen administration (Fig. 3C–E), consistent with basal CreERT2 activity. However, tdTomato expression alone does not necessarily indicate recombination at the *Slc10a4* locus. Indeed, immunostaining revealed preserved SLC10A4 expression in serotonergic neurons before tamoxifen treatment (Fig. 3F–H).

To quantify potential background recombination, we measured the ratio of SLC10A4-positive cells relative to NeuN-positive neurons within the dorsal raphe nucleus. Compared with Cre-negative controls (*Slc10a4*^*lox/ko*^*; Sert*^*wt/wt*^), experimental mice (Slc10a4-Sert-icKO) showed a detectable reduction in SLC10A4 expression prior to tamoxifen administration (Fig. 3I), consistent with baseline recombination due to CreERT2 leakiness. This reduction was statistically significant across experimental groups (Cre-negative controls, experimental mice before tamoxifen and experimental mice after tamoxifen; n = 5 per group; Kruskal–Wallis test (KW), H = 35.871, p < 0.0001; post hoc Mann–Whitney U test (MW), p < 0.001). In contrast, no significant differences were observed across rostrocaudal regions of the dorsal raphe (rostral: -4.48 to -4.60 mm, intermediate: -4.72 to -4.84 mm, caudal: -4.96 to -5.34 mm from Bregma; KW, H = 0.824, p = 0.66).

In contrast, tamoxifen-treated experimental mice exhibited complete loss of SLC10A4 expression throughout the rostrocaudal extent of the dorsal raphe (Fig. 3J). Statistical analysis confirmed a significant effect of genotype/treatment (n = 5 per group; KW, H = 36.273, p < 0.0001, and post-hoc MW test, p < 0.001) with no influence of rostrocaudal position, demonstrating efficient and uniform recombination across serotonergic nuclei.

### Adult serotonergic deletion of *Slc10a4* reduces extracellular serotonin levels in the medial prefrontal cortex (mPFC)

**Fig. 4:**
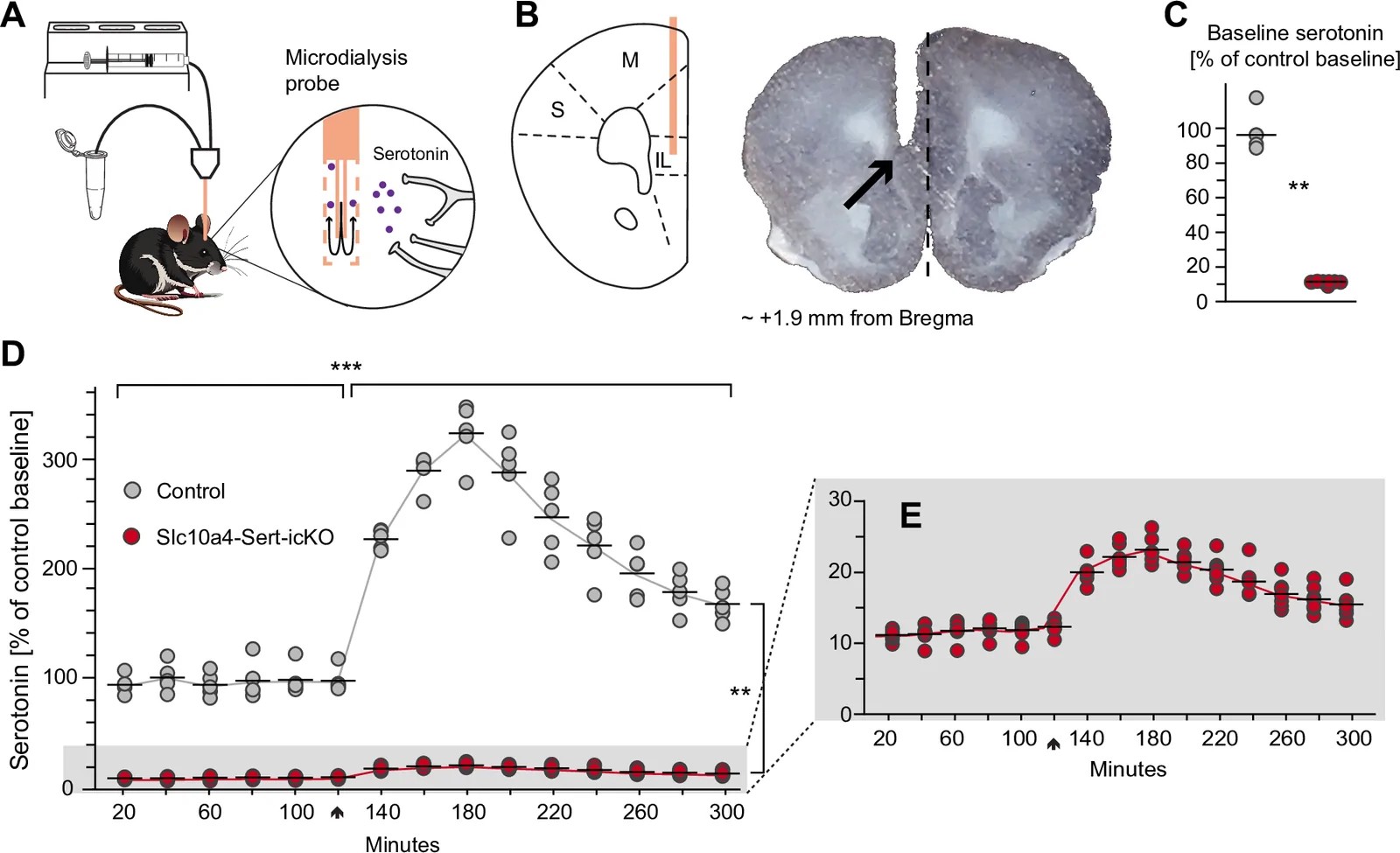
Reduced extracellular serotonin and blunted fluoxetine response in the mPFC of Slc10a4-Sert-icKO mice. **A** Microdialysis schematic. **B** Probe placement in the medial prefrontal cortex (mPFC; atlas view and representative coronal section showing probe placement (approximately +1.9 mm from Bregma, according to Paxinos and Franklin atlas; M, motor; S, sensory; IL, infralimbic). **C** Baseline extracellular serotonin levels expressed as percentage of control (individual values with median; **p < 0.01, Mann–Whitney U-Test). **D** The time course of mPFC serotonin levels before and after fluoxetine (20 mg/kg, i.p.) injection at 120 min indicated by the black arrow. Data is presented as scatter with mean, n = 5 control and n = 7 experimental mice, **p < 0.01; ***p < 0.001, repeated measures ANOVA. **E** Expanded post-fluoxetine response in Slc10a4-Sert-icKO mice (scatter with mean).

We performed in vivo microdialysis in the mPFC to determine whether adult deletion of *Slc10a4* in serotonergic neurons alters extracellular serotonin levels in projection areas (Fig. 4A). Correct placement of the microdialysis probe within the mPFC was confirmed after completion of the experiment (Fig. 4B). Baseline extracellular serotonin levels in the mPFC were significantly reduced in Slc10a4-Sert-icKO mice compared with control animals (Fig. 4C; **p < 0.01, Mann–Whitney U test), indicating that loss of SLC10A4 in serotonergic neurons leads to a sustained reduction in tonic serotonin availability at cortical target sites.

### Loss of serotonergic *Slc10a4* blunts fluoxetine-induced elevation of extracellular serotonin

We next examined the serotonergic response to pharmacological challenge with the selective serotonin reuptake inhibitor fluoxetine (20 mg/kg, i.p.). In control mice, fluoxetine administration induced a robust and sustained increase in extracellular serotonin levels in the mPFC, beginning shortly after drug injection and peaking within 60 minutes (Fig. 4D, gray dots). In contrast, Slc10a4-Sert-icKO mice displayed a markedly attenuated increase in serotonin levels following fluoxetine treatment (inset in Fig. 4D). Statistical analysis revealed a significant group × time interaction (n = 5 control and n = 7 experimental mice; **p < 0.01; ***p < 0.001; repeated measures ANOVA), demonstrating a reduced serotonergic responsiveness to SSRI treatment in experimental animals.

An expanded view of the post-fluoxetine time course in Slc10a4-Sert-icKO mice further illustrates the blunted serotonergic response relative to controls (Fig. 4E). Together, these data indicate that adult, serotonergic-specific deletion of *Slc10a4* leads to reduced basal extracellular serotonin levels in the mPFC and impairs the ability of fluoxetine to elevate serotonin.

### Adult serotonergic deletion of *Slc10a4* increases anxiety-like behavior

**Fig. 5:**
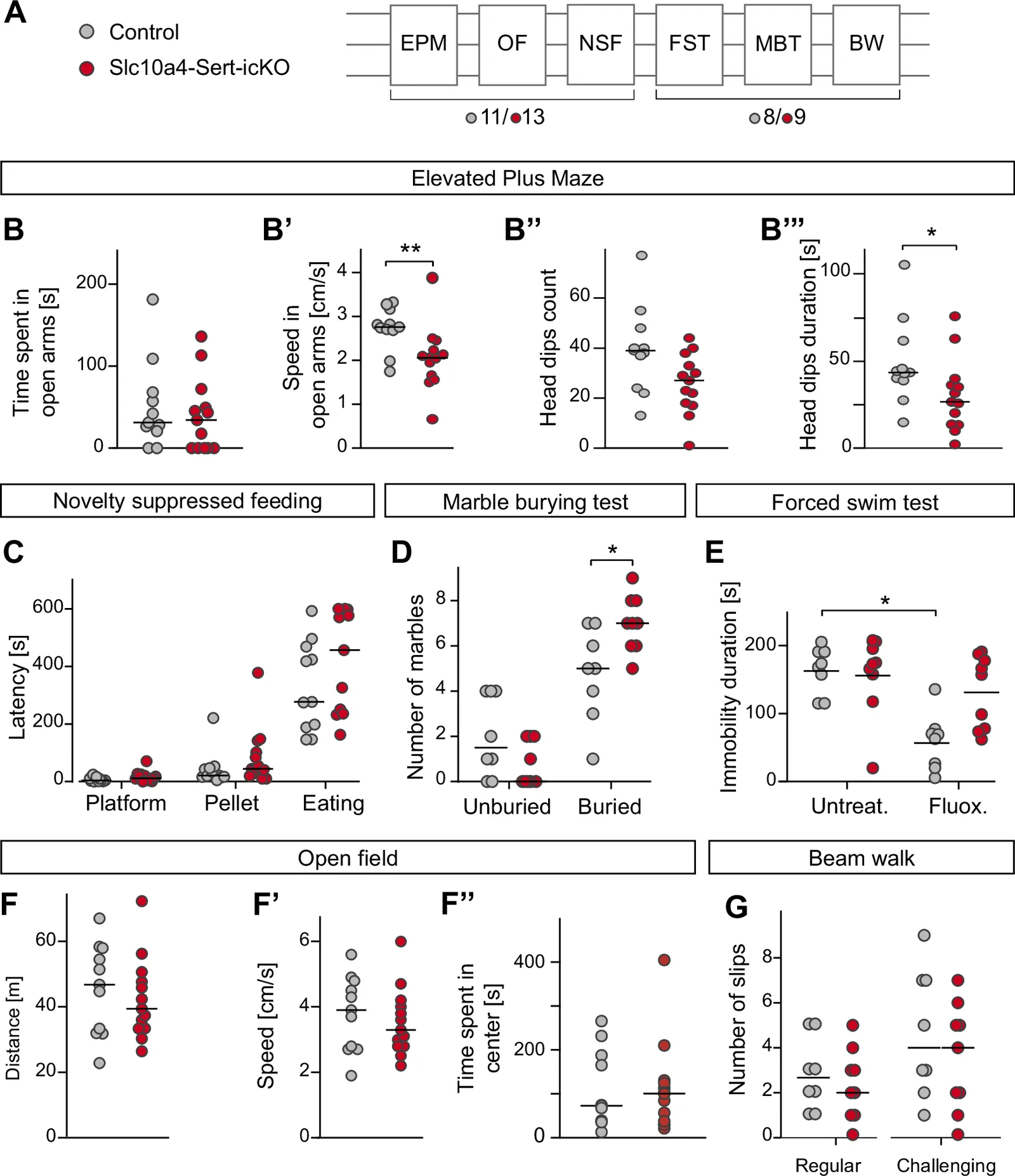
Behavioral effects of adult serotonergic deletion of *Slc10a4.* **A** Schematic overview of the behavioral test battery and number of animals analyzed per assay. Control mice are shown in gray and Slc10a4-Sert-icKO mice in red. **B**–**B**‴ Elevated plus maze performance, including time spent in the open arms (**B**), speed in the open arms (**B**’), number of head dips (**B**″), and head-dip duration (**B**‴). **C** Novelty-suppressed feeding test showing latency to enter the platform, contact the food pellet, and initiate feeding. **D** Marble burying test showing number of marbles buried versus unburied. **E** Forced swim test showing immobility duration at baseline (untreated) and following fluoxetine administration (20 mg/kg, i.p.). **F**–**F**″ Open field test showing total distance traveled (**F**), mean speed (**F**’), and time spent in the center of the open field (**F**”). **G** Motor coordination assessed by beam walk and challenging beam walk, shown as number of slips. Data are shown as individual values with median. Statistical analyses were performed using Mann–Whitney U tests. *p < 0.05, **p < 0.01.

Anxiety-like behavior was next evaluated using the elevated plus maze (EPM), novelty-suppressed feeding (NSF), and marble burying test (MBT). In the EPM (Fig. 5B), both groups spent a similar amount of time in the open arms (MW, p = 0.64), but Slc10a4-Sert-icKO mice exhibited reduced movement speed in the open arms (MW, p = 0.009). Although there was no difference in head dip counts (MW, p = 0.052), experimental animals had shorter head-dipping duration (MW, p = 0.02), when compared with control mice. In the NSF test (Fig. 5C), no significant difference was observed in latency to enter the platform (MW, p = 0.15), contact the food pellet (MW, p = 0.16) nor latency to eat (MW, p = 0.18). In the MBT (Fig. 5D), Slc10a4-Sert-icKO mice buried significantly more marbles than controls (MW, p = 0.021), consistent with increased anxiety-like behavior.

### Serotonergic *Slc10a4* deletion impairs antidepressant-like behavioral responses to fluoxetine

Depressive-like behavior and antidepressant responsiveness were assessed using the forced swim test (FST; Fig. 5E). Two-way repeated measures ANOVA on duration of immobility revealed a significant main effect of treatment (F(1,15) = 23.22, p = 0.00023) and a significant genotype × treatment interaction (F(1,15) = 8.96, p = 0.0091), with no significant main effect of genotype (F(1,15) = 3.16, p = 0.096). Fluoxetine markedly reduced immobility in control mice, whereas Slc10a4-Sert-icKO mice failed to respond to treatment, indicating that the lack of *Slc10a4* in serotonergic cells altered the response to the SSRI, a hallmark of depressive-like phenotypes in rodents.

### Adult serotonergic deletion of *Slc10a4* does not affect locomotor activity or motor coordination

To determine whether adult serotonergic deletion of *Slc10a4* affects general locomotion or motor performance, mice were first assessed in the open field (OF) and beam walk tasks (Fig. 5F, G). Slc10a4-Sert-icKO mice did not differ from control animals in total distance traveled or exploration patterns in the OF (Distance - MW, p = 0.56; Speed - MW, p = 0.56; Time spent in center - MW, p > 0.99), nor did they display deficits in motor coordination in either the beam walk or challenging beam walk tasks (MW, p = 0.66; MW, p = 0,44, respectively). These results indicate that deletion of *Slc10a4* in serotonergic neurons does not impair baseline locomotion or motor abilities.

## Discussion

In this study we present a cellular and molecular characterization of an inducible, serotonergic neuron–specific *Slc10a4* knockout model that reveals a critical role for *Slc10a4* in adult serotonergic neurotransmission, extracellular serotonin homeostasis, and affective behavior. By restricting gene deletion to mature serotonergic neurons, we demonstrate that loss of *Slc10a4* is sufficient to reduce cortical serotonin availability, blunt responsiveness to selective serotonin reuptake inhibition, and induce anxiety- and depression-related behavioral phenotypes, without confounding effects on locomotion or motor coordination. These findings identify *Slc10a4* as an essential regulator of serotonergic neurotransmission and extracellular serotonin homeostasis in the adult brain.

### Avoiding developmental compensation reveals a serotonergic-specific role of *Slc10a4*

Previous studies employing constitutive *Slc10a4* knockout mice reported altered monoamine content and synaptic phenotypes but also revealed extensive compensatory adaptations, particularly at the neuromuscular junction and within aminergic systems. These changes include reduced synaptic acetylcholine content, an increased readily releasable vesicle pool, and altered post-synaptic neuromuscular junction (NMJ) composition [29]. They also show decreased vesicular uptake efficiency and slower dopamine clearance [10], as well as reduced dopamine and acetylcholine content with increased dopamine turnover [12]. Such developmental compensation likely masks the primary function of *Slc10a4* in mature neural circuits. The inducible strategy used here was designed explicitly to circumvent this limitation by allowing *Slc10a4* deletion after serotonergic circuits have fully developed.

Our validation experiments confirmed efficient, spatially restricted recombination within serotonergic nuclei. Notably, adult serotonergic deletion of *Slc10a4* produced robust anxiety-like behavior and impaired antidepressant responsiveness, phenotypes that are absent or markedly attenuated in constitutive knockout models [10, 12], which reported only mild hypoactivity [10] or mild memory deficits [12]. This contrast strongly supports the conclusion that developmental compensation obscures the contribution of *Slc10a4* to serotonergic signaling and highlights the importance of temporally controlled genetic approaches for dissecting monoaminergic function.

### Serotonergic *Slc10a4* deletion in adult neurons and anxiety

Selective deletion of *Slc10a4* resulted in increased anxiety-like behaviors in the elevated plus maze and marble burying test, without affecting locomotor activity or motor coordination (Fig. 5B–G). The absence of deficits in open-field exploration and beam-walk performance indicates that the observed behavioral phenotypes are unlikely to result from nonspecific motor impairments and instead reflect altered affective processing [30].

Given the role of serotonin in the regulation of feeding behavior, it is also important to consider potential effects on appetite in the interpretation of the novelty-suppressed feeding test. However, we did not observe differences in body weight or food consumption following the fasting procedure (data not shown), suggesting that the behavioral effects are unlikely to be driven by altered feeding per se. Future studies using complementary assays will be important to further dissociate anxiety-related from feeding-related components of this behavior.

The increased marble-burying behavior in Slc10a4-Sert-icKO mice is consistent with heightened anxiety-related behavior linked to altered serotonergic signaling [31]. Also, it aligns with prior gene-expression studies associating *Slc10a4* with trait anxiety [32]. These converging findings support a role for *Slc10a4*-dependent vesicular monoamine handling in the regulation of anxiety-related behavior in the adult brain.

Previous work in genetically heterogeneous NIH-HS rats has identified *Slc10a4* as a candidate gene associated with trait anxiety, based on differential gene expression in animals selected for extreme behavioral phenotypes. These rats represent a highly recombinant population in which gene expression differences are correlated with anxiety-related traits, including performance in tasks that involve a significant component of associative learning, such as active avoidance paradigms [32].

At first glance, these findings may appear at odds with the present results, as increased *Slc10a4* expression was associated with higher anxiety in NIH-HS rats, whereas conditional deletion of *Slc10a4* in serotonergic neurons here leads to increased anxiety-like behavior. However, an important distinction lies in the nature of the behavioral assays employed. The NIH-HS studies include tasks with strong learning and memory components, whereas the present study primarily assesses innate anxiety-like behaviors. Given the established role of serotonergic and hippocampal circuits in both anxiety and learning processes, it is plausible that *Slc10a4* differentially modulates these domains.

In this context, our findings do not contradict but rather extend previous observations, providing causal evidence that *Slc10a4* contributes to the regulation of anxiety-related behavior, while also suggesting that its role may depend on the specific behavioral domain and underlying neural circuitry engaged.

### Impaired SSRI responsiveness links *Slc10a4* to antidepressant efficacy

In vivo microdialysis measurements revealed that *Slc10a4* deletion leads to a clear reduction in extracellular serotonin levels in the medial prefrontal cortex under baseline conditions, as well as a marked attenuation of the serotonergic response to fluoxetine (Fig. 4E). Whereas control mice displayed the expected robust elevation of extracellular serotonin following SSRI administration, Slc10a4-Sert-icKO mice showed a substantially blunted response. This reduced sensitivity to fluoxetine is consistent with the lack of antidepressant-like effects observed in the forced swim test (Fig. 5E), linking altered serotonergic dynamics to behavioral outcomes.

Because fluoxetine acts by inhibiting serotonin reuptake rather than directly promoting release, these findings suggest that intact presynaptic serotonergic function is required for full SSRI efficacy. However, given the limitations of microdialysis, our data do not allow us to determine whether this reflects alterations in vesicular loading, release probability, or other presynaptic mechanisms.

These findings highlight a functional interplay between plasma membrane and vesicular transport mechanisms in serotonergic neurotransmission. While selective serotonin reuptake inhibitors such as fluoxetine act at the level of the serotonin transporter (SERT) to block reuptake, their efficacy ultimately depends on the availability of releasable serotonin pools, which are determined by vesicular loading mechanisms. In this context, *Slc10a4* may represent a complementary component of this system, acting upstream of SERT-dependent processes. Disruption of vesicular function would therefore be expected to limit the effectiveness of reuptake inhibition, providing a potential explanation for the reduced SSRI responsiveness observed in Slc10a4-Sert-icKO mice. This raises the possibility that targeting both vesicular and membrane transport processes may represent a more effective strategy for modulating serotonergic signaling in affective disorders.

A mechanistic framework for *Slc10a4* function has previously been proposed, in which it facilitates vesicular anion flux and thereby supports the electrochemical gradients required for efficient neurotransmitter loading [10]. According to this model, loss of *Slc10a4* could impair aminergic vesicular function, as suggested by previous studies in peripheral synapses [29]. While our findings are consistent with this hypothesis, they do not directly test vesicular loading or release mechanisms in central serotonergic neurons. Future studies using stimulus-evoked paradigms or serotonin-releasing agents will be required to directly assess presynaptic release mechanisms and further delineate the contribution of *Slc10a4* to vesicular versus release-related processes.

Nevertheless, the observed reduction in baseline extracellular serotonin and the blunted pharmacological response to fluoxetine provide in vivo evidence that *Slc10a4* is required for normal serotonergic signaling dynamics, and may represent a previously underappreciated determinant of antidepressant responsiveness.

### Limitations

A limitation of this study is the inherent leakiness of inducible CreERT2 systems, which can produce low levels of recombination in the absence of tamoxifen [33]. Consistent with previous reports using SertCreERT2 drivers, we detected minimal baseline recombination prior to tamoxifen administration, which was insufficient to abolish SLC10A4 expression in serotonergic neurons. To control for effects of Cre expression and tamoxifen exposure, all comparisons were made to tamoxifen-treated *Slc10a4*^ko/wt^ SertCreERT2-positive littermates. Complete loss of SLC10A4 was observed only after tamoxifen administration (Fig. 3J), supporting the conclusion that the observed phenotypes result specifically from adult serotonergic deletion of *Slc10a4*.

In the present study tamoxifen-induced recombination was performed at 8–10 weeks of age, corresponding to young adulthood rather than fully mature adult stages. While this approach avoids developmental compensation associated with constitutive models, it remains possible that serotonergic circuit maturation and plasticity at this stage may influence the observed phenotypes. Future studies examining *Slc10a4* and aging will be important to determine the generalizability of our findings across the lifespan.

An additional limitation is that all experiments were conducted in male mice. Given known sex differences in serotonergic signaling and affective behavior, the extent to which these findings generalize to females remains to be determined. Future studies incorporating both sexes will be important to assess potential sex-specific effects of *Slc10a4* deletion.

Also, we note that the forced swim test, a known acute stressor, was performed prior to marble burying and beam-walk assessments. However, a prolonged interval (approximately one month) separated the forced swim test from the marble burying test, followed by a 3-day interval before beam-walk testing. These intervals substantially reduce the likelihood of carryover stress effects influencing subsequent behavioral performance. Nevertheless, we cannot fully exclude subtle effects of prior stress exposure.

### Broader implications and future directions

Beyond its role in serotonergic neurotransmission, accumulating evidence suggests that *Slc10a4* may represent a more general regulator of vesicular function across multiple cell types and aminergic systems. Previous studies have implicated *Slc10a4* in a range of neuropsychiatric and neurological conditions, including altered sensory gating relevant to schizophrenia [13], increased seizure susceptibility [14], and dopaminergic dysfunction linked to Parkinson’s disease [15]. In addition, reduced SLC10A4 expression in the human trans-entorhinal cortex correlates with Alzheimer’s disease severity [11], suggesting that impaired vesicular monoamine handling may contribute to neurodegenerative pathology.

Importantly, deficits associated with *Slc10a4* loss are not restricted to neurons. Mast cells lacking *Slc10a4* exhibit markedly reduced granule content, including monoamines and other bioactive substances [8], supporting a conserved role for *Slc10a4* in vesicular filling across secretory cell types. Similarly, cholinergic synaptic vesicles isolated from *Slc10a4* knockout mice contain fewer neurotransmitter quanta [29], further reinforcing the concept that *Slc10a4* modulates vesicular loading efficiency rather than neurotransmitter synthesis or transporter expression.

In addition to genetic and functional evidence, emerging epigenomic data further support a role for *Slc10a4* in long-term brain regulation. Recent analyses of age-associated epigenomic signatures identified *Slc10a4* among the top genes potentially regulated by age-dependent chromatin changes, implicating cell signaling pathways sensitive to aging processes [34]. This observation raises the possibility that SLC10A4 expression and function may be dynamically modulated across the lifespan, linking vesicular monoamine handling to age-related changes in affective regulation and vulnerability to neuropsychiatric disorders.

Together with the present findings, these observations position *Slc10a4* as a central component of vesicular monoamine homeostasis whose dysfunction may impact affective behavior, antidepressant responsiveness, and broader aspects of brain and peripheral signaling. Future studies employing cell-type- and circuit-specific conditional manipulations of *Slc10a4* in dopaminergic, cholinergic, and noradrenergic systems will be essential to determine whether analogous mechanisms operate across aminergic networks and to assess the therapeutic potential of targeting vesicular loading processes in mood and psychiatric disorders.

## Data Availability

No new sequence, structural, or large-scale datasets were generated in this study. Source data are available from the corresponding authors upon reasonable request.
